# Traditional Chinese Medicinal Leech Induces Apoptosis and Autophagy in Glioblastoma by SGK1/Caspase‐3 and PI3K/AKT/mTOR Pathway

**DOI:** 10.1002/cns.70683

**Published:** 2025-12-01

**Authors:** Shaohua Wu, Yaya Zhou, Zhuan Pei, Yang Wang, Zuping Zhang

**Affiliations:** ^1^ Department of Parasitology, Xiangya School of Basic Medical Sciences Central South University Changsha Hunan People's Republic of China; ^2^ Institute of Integrative Medicine, Department of Integrated Traditional Chinese and Western Medicine, Xiangya Hospital Central South University Changsha Hunan People's Republic of China; ^3^ Department of Neurology, the Third Xiangya Hospital Central South University Changsha Hunan People's Republic of China; ^4^ Laboratory for Interdisciplinary Science of TCM, Xiangya Hospital Central South University Changsha Hunan People's Republic of China

**Keywords:** apoptosis, autophagy, glioblastoma, leech

## Abstract

**Background:**

Glioblastoma (GBM) represents the most lethal form of high‐grade glioma, with current therapeutic options proving largely ineffective. Consequently, there is an urgent need for novel treatment strategies. Recent studies have indicated that the medicinal leech exhibits notable anticancer properties. However, the precise mechanisms underlying these effects remain to be elucidated.

**Methods:**

To investigate the impact of leech drug‐containing serum (LDS) on the proliferation, migration, and invasion of GBM cells, a series of assays including CCK‐8, ethynyl deoxyuridine (EdU), colony formation, scratch, and transwell assays were employed. Additionally, the apoptosis and autophagy of GBM cells were analyzed using flow cytometry, monodansylcadaverine staining and the immunofluorescence assay. Transcriptomic sequencing of the cells was conducted to identify differentially expressed genes. In vivo, anticancer activity was assessed by developing tumor xenograft models. Western blot analysis was utilized to identify proteins associated with apoptosis and autophagy.

**Results:**

Leech drug‐containing serum (LDS) significantly inhibited proliferation, migration, and invasion. Furthermore, it induced autophagy and apoptosis in GBM. Differential gene enrichment analysis and pathway validation indicated that LDS exerts anti‐GBM effects by modulating the PI3K/AKT pathway. Leech extracts effectively inhibit the growth of GBM in a subcutaneous xenograft tumor model.

**Conclusion:**

The leech‐derived compounds may induce apoptosis and autophagy in GBM by modulating the PI3K/AKT/mTOR signaling pathway, without eliciting significant adverse effects, thereby presenting itself as a promising therapeutic agent.

## Graphical abstract

Schematic representation of the underlying molecular mechanisms by which leech mediates apoptosis and autophagy through SGK1/Caspase‐3 and PI3K/AKT/mTOR pathways to inhibit glioblastoma.

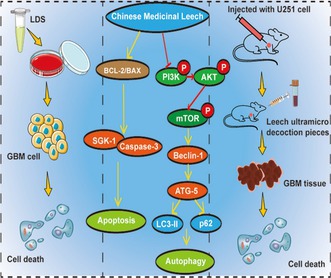

## 1 Introduction

Glioblastoma (GBM), the most prevalent and aggressive primary brain tumor, is characterized by an exceptionally poor prognosis and high mortality. Despite multimodal therapeutic interventions including maximal safe resection, radiotherapy and temozolomide‐based chemotherapy, the median progression‐free survival remains dismal, with minimal improvements in overall survival over the past decade [1]. This critical unmet medical necessity underscores the imperative for innovative treatment strategies.

Whether employed independently or alongside Western therapies, TCM, a crucial element of complementary and alternative medicine, demonstrates efficacy in treating a wide range of diseases. TCM has garnered significant attention in recent years as a potential cancer treatment, attributed to its distinctive characteristics, including its multicomponent and multitarget approach, as well as its relative safety profile [2]. Historically, in clinical practice, the medicinal leech was initially recognized for its antithrombotic effects, as it could continuously extract blood upon attaching to human skin [3, 4]. The use of leech in Chinese traditional medicine is documented in “The Shengnong Bencao Jing”, a text dating back over 2000 years [5]. According to the Chinese Pharmacopeia, the leech species predominantly utilized in TCM include Whitmania pigra Whitman, Hirudo nipponica Whitman, and Whitmania acranulata, although the latter two species are relatively rare [6]. Hirudin, a potent thrombin inhibitor, is secreted by the salivary glands of leeches [7]. This compound has been applied in the treatment of various human tumors, such as epithelioid carcinoma, adenocarcinoma, lung carcinoma, cholangiocarcinoma, and breast adenocarcinoma [8]. Hirudo extracts modulated the expression of methyltransferases to inhibit proliferation and induce apoptosis of liver cancer [9]. Meanwhile, hirudo drug‐containing plasma and serum inhibited the proliferation and migration of human pancreatic cancer, which may be related to the decreased expression of VEGF [10]. Previous studies have established that hirudin exhibits multiple modes of action against GBM, encompassing anti‐inflammatory effects, modulation of cell proliferation, induction of apoptosis, and inhibition of cell adhesion, migration, invasion, metastasis, and oxidative stress. However, the broader impact of leech‐derived compounds, beyond just hirudin, on GBM and their underlying mechanisms necessitates further investigation.

This study explores the anti‐GBM properties of leech, revealing a significant suppression of proliferation, migration, and invasion in GBM cells. Further investigation into the mechanisms revealed that leech drug‐containing serum (LDS) induces apoptosis and autophagy in GBM cells by modulating the Serum and glucocorticoid‐regulated kinase‐1 (SGK1)/Caspase‐3 pathway and the phosphatidylinositol 3‐kinase (PI3K)/Akt/mammalian target of rapamycin (mTOR) pathway, respectively. These findings were corroborated in a subcutaneous implantation mouse model. Additionally, leech exhibits a favorable safety profile. Based on these results, leech may represent a less cytotoxic and more effective chemotherapeutic agent for the treatment of GBM.

## 2 Materials and Methods

### 2.1 Cell Line Selection and Cell Culture

The U251 and C6 cell lines were selected for our study. U251is a widely used cell line for WHO grade IV glioblastoma, which harbors classic mutations including PTEN deletion and EGFR amplification that mimic human GBM pathophysiology. C6 cells are derived from chemically induced rat gliomas, which are of great significance in the study of glioma.

Dulbecco's Modified Eagle Medium with high glucose, supplemented with 10% fetal bovine serum (FBS, Cellmax, China) and 1% penicillin/streptomycin (Gibco, USA), was utilized to maintain the cells at 37°C in a humidified atmosphere containing 5% CO_2_.

### 2.2 Preparation of Leech Drug‐Containing Serum

Leech ultramicro decoction pieces, produced by Hunan Chunguang Jiuhui Modern Chinese Medicine Co. LTD (Product Lot number: 81221003), were procured from Xiangya Hospital, Central South University. Healthy male Sprague–Dawley (SD) rats (8w, 200‐220 g) were employed in the experiments, and all procedures were approved by the Committee on the Care and Use of Animals at Central South University. After an acclimatization period of approximately 1 week, 20 rats were randomly assigned to either a blank serum (BS) group or an LDS group. The dosage of leech ultramicro decoction pieces was calculated based on the body surface area of the rats. Each rat received a daily gavage for 7 days at a dosage of 26.5 g/kg. Rats in the blank group were administered normal saline. One hour following the final administration [11]. the rats were anesthetized using isoflurane. Blood was collected from the abdominal aorta and allowed to stand for 1 h. Subsequently, the blood samples were centrifuged to separate the serum. Sera samples from 10 rats were pooled and then inactivated by heating at 56°C for 30 min, followed by filtration through a 0.22 μm filter.

### 2.3 Analysis of LDS Compounds

We used a negative ion mode of liquid chromatography–tandem mass spectrometry (LC–MS/MS) to detect main active ingredients. The samples were prepared in a solvent mixture of 0.1% methanol/water and 0.1% formic acid/acetonitrile. Separation was performed on an LCMS‐8050 system with a flow rate of 0.4 mL/min and an injection volume of 10 μL. For mass analysis, compounds were ionized in negative mode using a triple quadrupole mass spectrometer.

### 2.4 Cell Counting Kit‐8 (CCK‐8) Assay

Cell viability was evaluated using the CCK‐8 assay (Vazyme, China). After exposure to varying serum concentrations (5%, 10%, and 20%) for either 24 or 48 h, CCK‐8 solution was added to the cells. The OD values of the wells were measured using a microplate reader at a wavelength of 450 nm.

### 2.5 Plate Clone Formation Assay

Initially, 1000 cells were seeded and incubated overnight. Two experimental groups, the LDS group and the BS group, were established, with the culture medium being replaced every 2 days. Cells were cultured until small white specks became visible at the bottom of the plate. The colonies were subjected to two rounds of washing with phosphate‐buffered saline, followed by fixation with 4% paraformaldehyde. Subsequently, 1 mL of crystal violet solution was applied to each well [12]. The residual dye solution was gently removed by rinsing with flowing water, followed by drying at ambient temperature. Images were captured, and colonies containing more than 50 cells were enumerated.

### 2.6 Wound Healing Assay

Cells were incubated overnight. Following the creation of scratches in the center of the tissue culture plates, the samples were subjected to an additional 48 h of treatment. Photographs were taken at specific intervals. The rate of scratch non‐healing was quantified using ImageJ software.

### 2.7 Invasion Assay

The complete medium was added to the wells of a 24‐well plate, and U251 and C6 cells (1 × 10^4^ cells per well, without FBS) were introduced into the transwell inserts, which were coated with Matrigel (Corning Biocoat, USA). Subsequently, the transwell inserts were removed and fixed in 4% formaldehyde for 15 min. The cells were then stained with 0.1% crystal violet and washed three times with phosphate‐buffered saline. Five visual fields per well were photographed using a microscope (Leica, USA), and the number of invading cells was counted.

### 2.8 DNA Synthesis Assay

In accordance with the provided protocol, the ethynyl deoxyuridine (EdU) Cell Proliferation Kit (Vazyme, C0078) was employed. After a 48‐h incubation period with 5%, 10%, and 20% LDS, EdU was administered to each well. Following an additional 2 h, the cells were fixed, permeabilized, and stained. Fluorescence microscopy (Leica, USA) was utilized for image acquisition.

### 2.9 Apoptosis Assay

Apoptosis was quantified using the Apoptosis Detection Kit (Beyotime, C1062), and the apoptosis rate was determined via flow cytometry (LSRFortessa SORP, BD, San Jose, CA, USA).

### 2.10 Immunofluorescence (IF) Assay

Cells were seeded and cultured under standard conditions. After 48 h of exposure to a medium containing either BS or LDS, the cells were subjected to fixation, permeabilization, and blocking procedures. Subsequently, the cells were incubated with a primary antibody specific for LC3B (Proteintech, 14,600‐1‐AP, 1:200 dilution). On the following day, a diluted secondary antibody (Donkey‐anti‐rabbit Cy3, Jakson, 711‐165‐152) was applied for 1 h, followed by a 10‐min incubation with DAPI solution (Solarbio, C0065, 1:1000 dilution). Finally, the coverslips were sealed, and images were acquired using a fluorescence microscope (ZEISS, Germany).

### 2.11 Monodansylcadaverine (MDC) Staining

A total of 2 × 10^5^ cells were cultured. Following various treatments, MDC (Beyotime, C3018) was administered to the cells. Subsequently, the cells were washed with assay buffer, and images were captured using a fluorescence microscope (Leica, USA).

### 2.12 Western Blot Assay

RIPA buffer (Beyotime, P0013B) was utilized to lyse the glioma cells. Protein concentration was determined using the bicinchoninic acid assay kit (Thermo, 23,227). Proteins were isolated via sodium dodecyl sulfate‐polyacrylamide gel electrophoresis, followed by electrophoretic transfer onto polyvinylidene difluoride membranes. Following the blocking step, the membranes were maintained overnight at 4°C with the following antibodies: β‐Actin (CST, #3700, 1:1000), GAPDH (Servicebio, GB11002, 1:1000), LC3B (Proteintech, 14,600‐1‐AP, 1:1000), ATG5 (Huabio, ET611‐38, 1:1000), p62 (Huabio, HA721171, 1:2000), Beclin1 (Huabio, HA721216, 1:2000), AKT (CST, #9272, 1:1000), phospho‐AKT (CST, 4060, 1:2000), mTOR (Abclonal, A2445, 1:2000), phospho‐mTOR (Abclonal, AP0049, 1:2000), Caspase‐3 (CST, #14220, 1:1000), BCL‐2 (Huabio, ET702‐53, 1:2000), BAX (Abclonal, A19684, 1:2000), SGK1 (Proteintech, 28,454‐1‐AP, 1:2000). The subsequent day, the membranes were incubated with the secondary antibody. Thereafter, enhanced chemiluminescence reagents were employed to visualize the proteins. Images were then captured, and the intensity of the protein bands was analyzed.

### 2.13 RNA Sequencing

RNA extraction was performed using the TRIzol reagent (Takara, Japan). The purity and integrity of the RNA were evaluated using a NanoPhotometer and an Agilent 2100 Bioanalyzer, respectively. NEB libraries were constructed using Illumina technology. The Database for Annotation, Visualization, and Integrated Discovery version 6.8 (https://david.ncifcrf.gov/) was employed to conduct an analysis of the Kyoto Encyclopedia of Genes and Genomes (KEGG) and Gene Ontology (GO) pathways. RNA sequencing was performed by Novogene Corporation (Beijing, China).

### 2.14 Tumor Xenograft Model

For the xenograft experiments, U251 cells were subcutaneously implanted into the right flanks of mice. Subsequently, these mice were randomly divided into four groups (*n* = 6 per group). The treatment regimen involved administering temozolomide (TMZ) at a dose of 50 mg/kg every 3 days, or leech ultramicro at doses of 80 mg/kg and 160 mg/kg daily, over a period of 3 weeks. The dosages of leeches were chosen according to the fourth edition of Pharmacological Experimental Methodology and previous studies [13]. Body weight and tumor size were measured every other day.

### 2.15 Hematoxylin–Eosin (HE) Staining

Slides were baked at 60°C for 1 h, dewaxed in xylene I/II (15 min each), and rehydrated through a graded ethanol series (100%–95%–75%, 5 min each). After rinsing with distilled water (5 min), hematoxylin staining was performed (6 min), followed by differentiation (0.5% acid‐alcohol, 3 s) and bluing (running water, 10 min). Eosin staining (1 min) was then applied, and slides were dehydrated in an ethanol series (75%–95%–100%), cleared in xylene (10 min), mounted with neutral resin, and imaged under a microscope.

### 2.16 Statistical Analysis

Data are shown as mean ± standard deviation (SD) from at least three independent experiments. All statistical results were analyzed and visualized by GraphPad Prism 8.0. Shapiro–Wilk test was used to evaluate normality. Data conforming to normal distribution were compared using Student *t*‐test or one‐way ANOVA, while those with non‐normal distribution were tested using Mann–Whitney *U*‐test or Kruskal–Wallis followed by Dunn's multiple comparisons. A *p*‐value of less than 0.05 was deemed statistically significant.

## 3 Results

### 3.1 Quality Control of LDS

Qualitative analysis of characteristic peaks of LDS was performed using UPLC‐MS/MS. Compound identification was achieved by comparing the retention time, MS1, and MS2 of compound peaks with standards. The LC/MS results showed that the LDS contained higher leech active ingredients compared with the BS, which contained hippuric acid, formylanthranilic acid, N‐(3‐Methylbut‐2‐EN‐1‐YL)‐9H‐purin‐6‐amine, and so on (Figure S1).

### 3.2 Leech Drug‐Contained Serum Inhibits the Growth of GBM Cells

**FIGURE 1:**
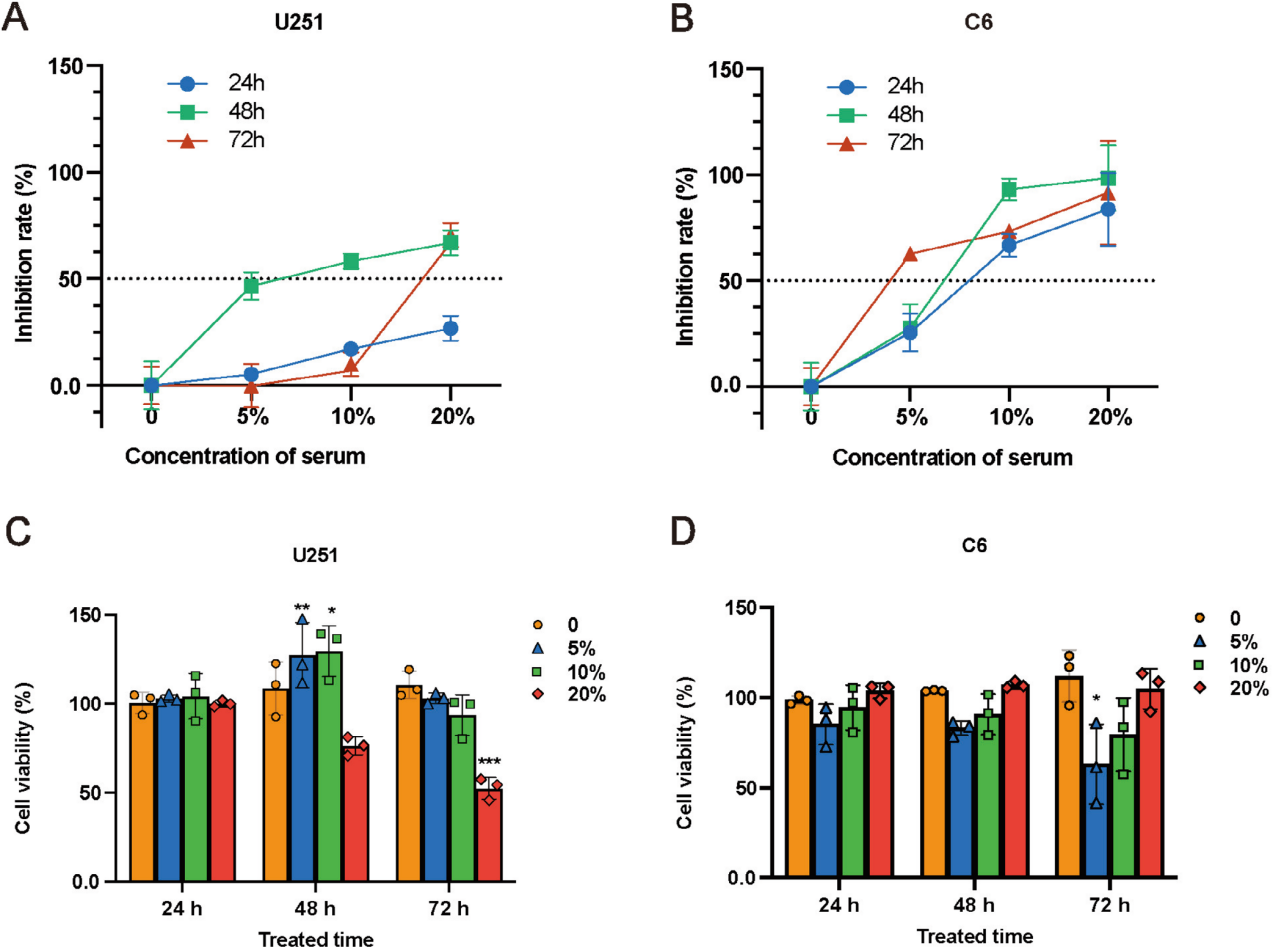
LDS inhibits cell growth in GBM cells. (A, B) Cell inhibition rate analysis using a CCK8 assay of GBM cell lines after treatment with various concentrations of LDS or vehicle alone for the indicated time period. (C, D) Cell viability analysis using a CCK8 assay of GBM cell lines after treatment with various concentrations of BS or vehicle alone for the indicated time period. Data are expressed as mean ± SD (*n* = 3). *p < 0.05, **p < 0.01, ***p < 0.001 versus control. GBM, glioblastoma; LDS, leech drug‐containing serum.

**FIGURE 2:**
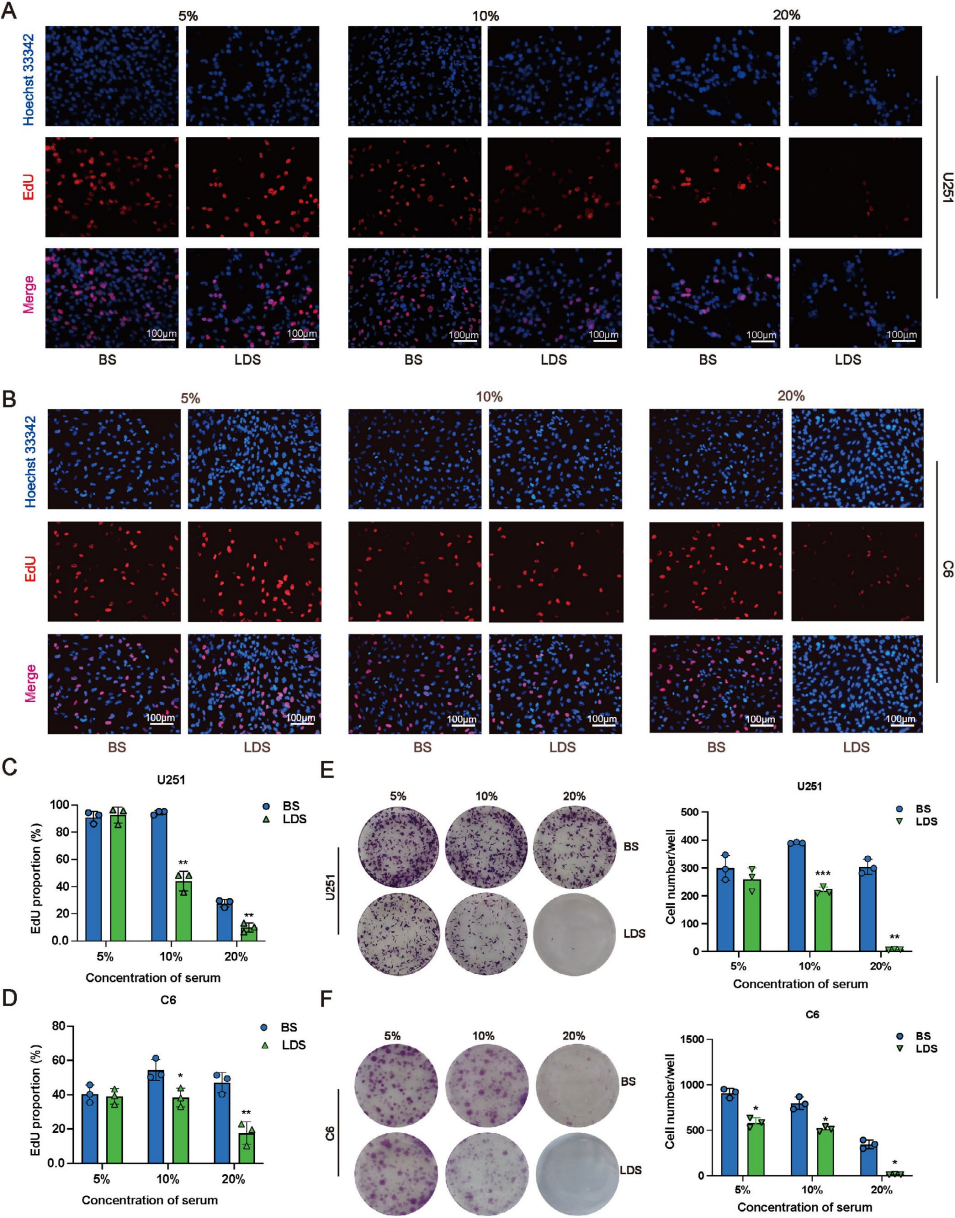
LDS inhibits cell proliferation in GBM cells. (A–D) LDS inhibited DNA synthesis in (A, C) U251 cells and (B, D) C6 cells at 48 h, which was analyzed by EdU incorporation. Scale bar = 100 μm. (E) LDS inhibited colony formation of U251 cells. (F) LDS inhibited colony formation of C6 cells. Data are expressed as mean ± SD (*n* = 3). *p < 0.05, **p < 0.01, ***p < 0.001 versus control. GBM, glioblastoma; LDS, leech drug‐containing serum.

Various assays were employed to evaluate the potential of LDS in inhibiting the proliferation of GBM. The CCK‐8 assay revealed that at concentrations of 10% and 20%, LDS achieved a suppression rate exceeding 50% on U251 and C6 cells after 48 h (Figure 1A–D). Additionally, the EdU assay was utilized to quantify the inhibitory effect of LDS on the proliferation of U251 and C6 cells, demonstrating a significant reduction in DNA synthesis at the same concentrations, and the inhibition rate of U251 cells was more than 50% (Figure 2A–D). Furthermore, the plate colony formation assay indicated that LDS treatment led to a decrease in the number of colonies formed by U251 and C6 cells (Figure 2E,F).

### 3.3 LDS Could Inhibit Migration and Invasion of GBM Cells

**FIGURE 3:**
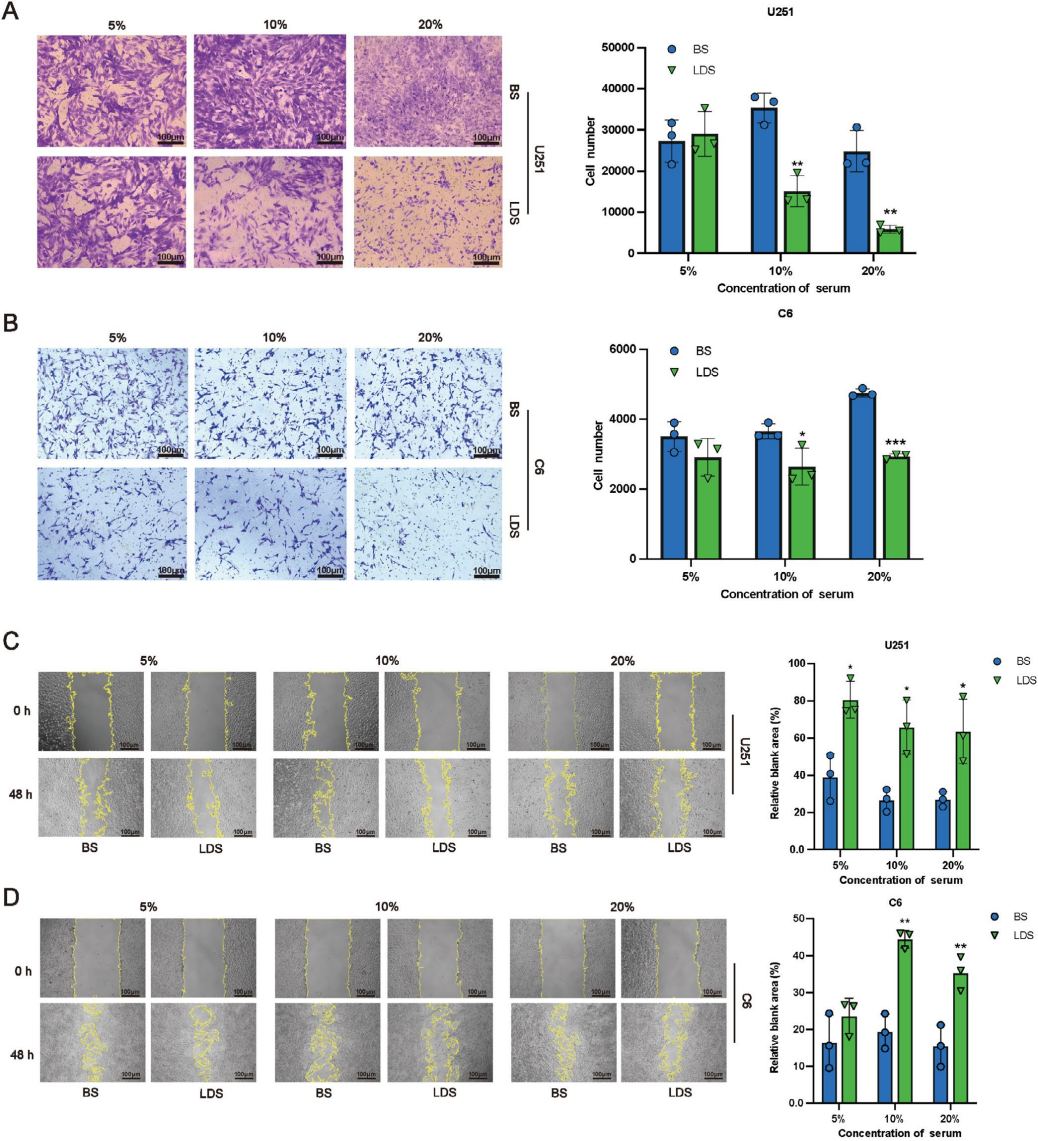
LDS inhibits cell migration and invasion in GBM cells. (A, B) Transwell assays showed LDS inhibited the invasion ability of U251 cells (A) and C6 cells (B). (C, D) Wound healing assay showed LDS inhibited the migration ability of U251 cells (C) and C6 cells (D). Scale bar = 100 μm. Data are expressed as mean ± SD (*n* = 3). *p < 0.05, **p < 0.01, ***p < 0.001 versus control. GBM, glioblastoma; LDS, leech drug‐containing serum.

The migration and invasion capabilities of tumor cells serve as critical indicators for assessing the aggressiveness of malignancies in vitro. Studies have reported that leech and hirudin inhibit the migration and invasion of human retinoblastoma and lung cancer cells. The ability of LDS to inhibit the migration and invasion of GBM was evaluated using wound healing and transwell assays. LDS significantly inhibited the invasion of U251 cells and C6 cells, and the inhibition rate of U251 was more than 50% (Figure 3A,B). The migration of U251 and C6 cells was obviously inhibited by more than 50% (Figure 3C,D). Overall, these findings support the hypothesis that LDS impedes the tumorigenic potential of GBM cells by obstructing their capacity to migrate, invade and proliferate.

### 3.4 LDS Induces Apoptosis and Autophagy in GBM Cells

**FIGURE 4:**
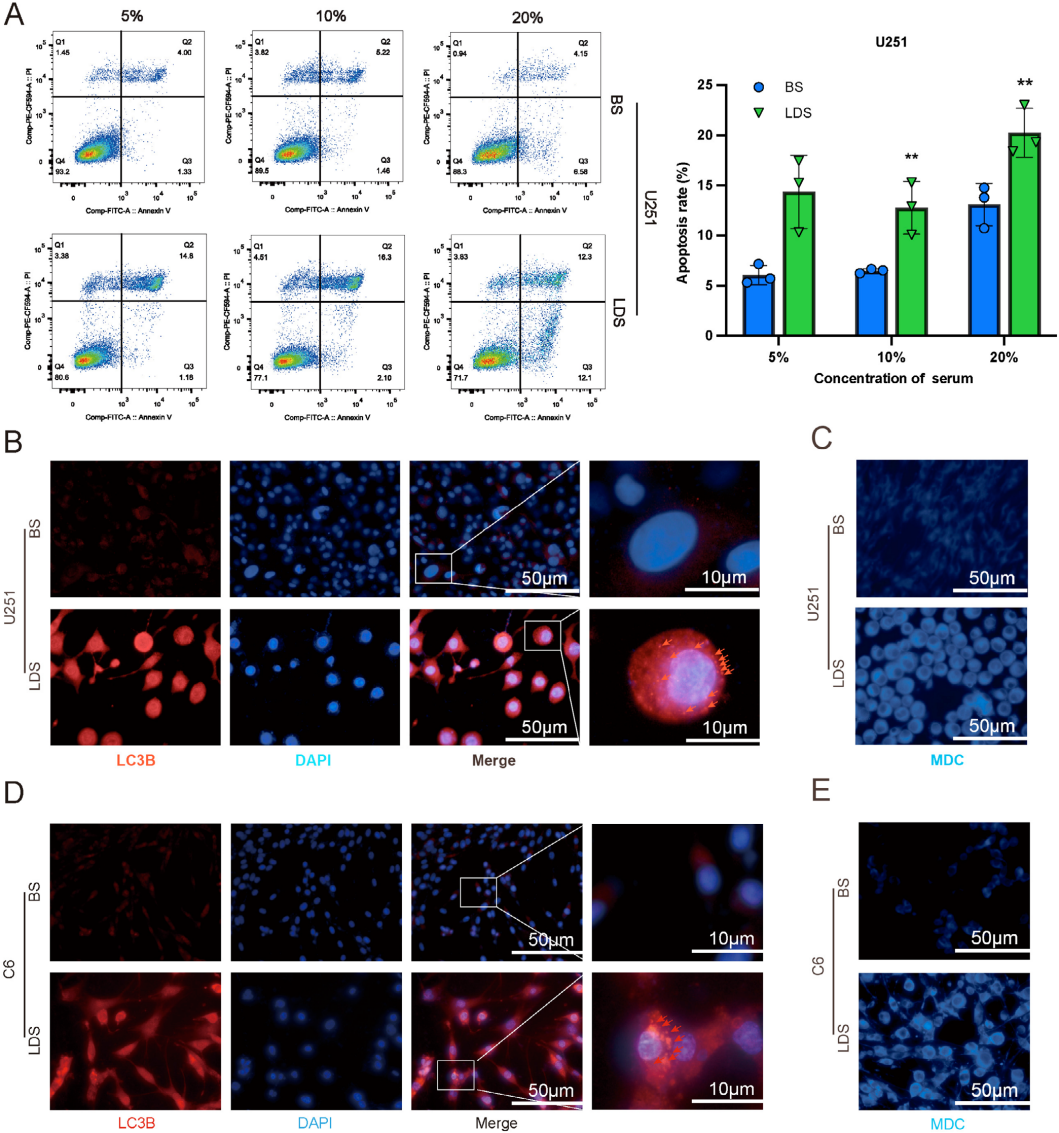
LDS induces cell apoptosis and autophagy in GBM cells. (A) Flow cytometry using PI/Annexin V‐FITC double stain showed LDS induced apoptosis of U251 at 48 h. (B–E) 10% LDS‐induced autophagy activity was determined with monodansylcadaverine (MDC) staining or IF staining for LC3 puncta in U251 cells (B, C) and C6 cells (D, E) at 48 h. Scale bar = 50 μm. Data are expressed as mean ± SD (*n* = 3). *p < 0.05, **p < 0.01, ***p < 0.001 versus control. GBM, glioblastoma; LDS, leech drug‐containing serum.

Recent studies have indicated that leech and hirudin possess the ability to induce apoptosis in tumor cells [14, 15]. After incubation with LDS, U251 and C6 cells were stained with PI and Annexin‐V‐FITC, followed by flow cytometry analysis to evaluate apoptosis. The results demonstrated a significantly higher proportion of apoptotic cells in the LDS‐treated group compared to the BS group (Figure 4A). To determine whether LDS can also induce autophagy in GBM cells, MDC staining and IF assays were employed. After 48 h of treatment with 10% LDS, a significant increase in distinct acidic vesicles was observed in U251 and C6 cells, with over 80% of the cells testing positive for MDC staining (Figure 4C,E). Immunofluorescence microscopy was employed to visualize LDS‐induced LC3 puncta in U251 and C6 cells, which further corroborated the activation of autophagic activity. In contrast, LDS‐treated U251 and C6 cells displayed prominent electron‐dense lysosomal structures (Figure 4B,D). Collectively, these findings indicate that LDS treatment stimulates autophagic flux.

### 3.5 Differential Expression Profiles of mRNAs in LDS‐Treated GBM Cells

**FIGURE 5:**
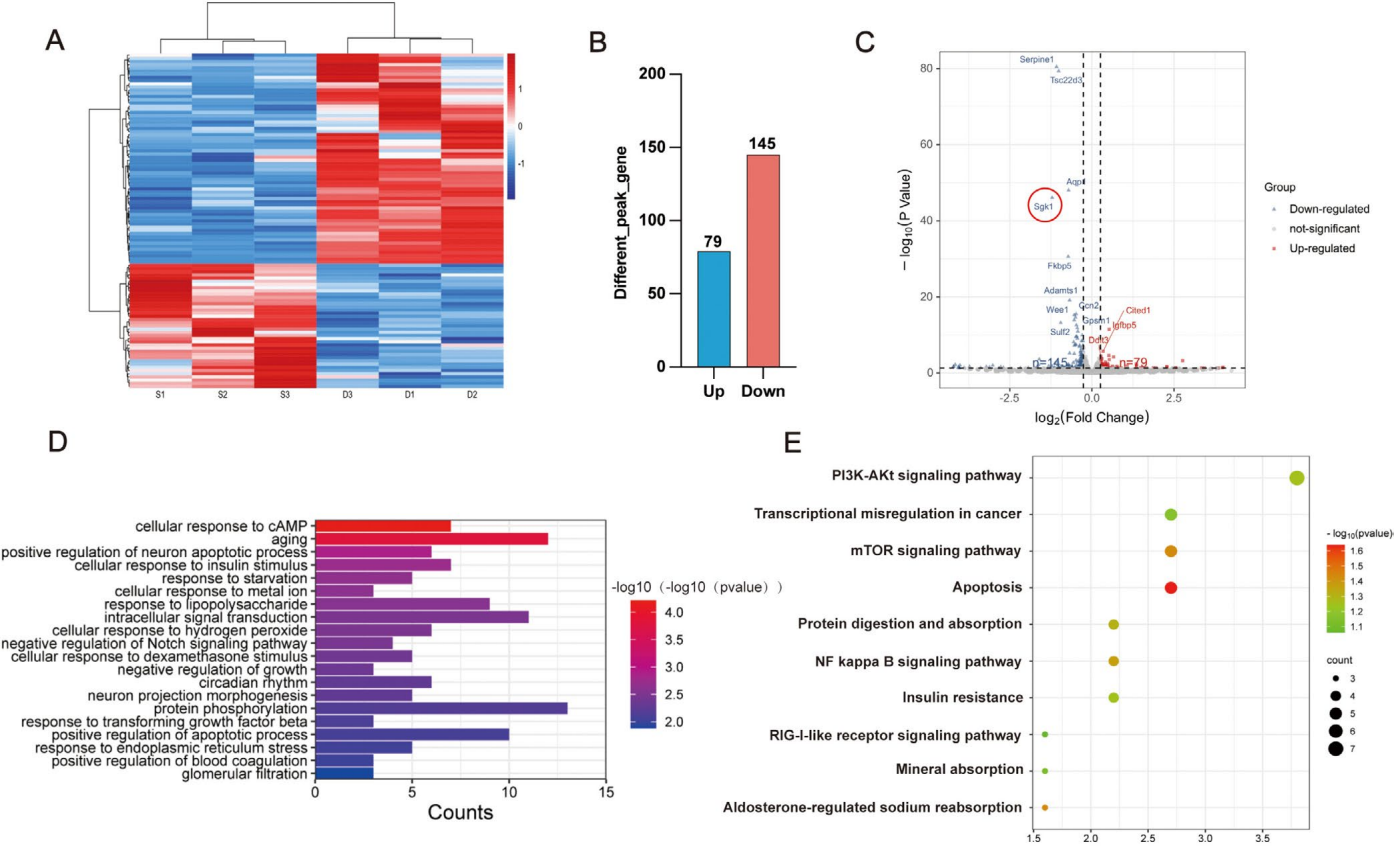
LDS induces significant changes of gene expression profile in C6 cells. (A) Heatmap of differentially expressed genes of C6 cells treated with 10% LDS for 48 h and visualized by Hiplotpro. (B) Amount of differentially expressed genes of C6 cells treated with 10% LDS for 48 h represented by a bar chart. (C) Visualization of differential genes by volcano plot of C6 cells treated with 10% LDS for 48 h. Red points for up‐regulated genes, blue for down‐regulated genes, and gray for unchanged genes (|FoldChange|≧ 1.25, *p* < 0.05). (D) Top 20 Histogram of GO enrichment analysis of differentially expressed genes. (E) Bubble chart of KEGG enrichment analysis of differentially expressed genes. Data are expressed as mean ± SD (*n* = 3). *p < 0.05, **p < 0.01, ***p < 0.001 versus control. GBM, glioblastoma; LDS, leech drug‐containing serum.

RNA was extracted and employed for RNA‐Seq analysis. Relative to the BS group, 79 genes exhibited upregulation, while 145 genes were downregulated in the LDS group. Differential gene expression was determined using threshold values of |Fold Change| ≥ 1.25 and *p* < 0.05 (Figure 5A–C). GO enrichment analysis revealed that the primary differentially regulated genes were associated with apoptosis, signal transduction, aging, and the regulation of apoptotic processes (Figure 5D). Furthermore, KEGG pathway analysis indicated that the LDS group significantly affected the PI3K/AKT signaling pathway, the mTOR signaling pathway, and apoptosis (Figure 5E). The serine/threonine kinase SGK1 was originally cloned from mammary tumor cells [16]. It has been implicated in the inhibition of apoptosis protection across various cancers, including breast cancer, colonic tumors, and prostate cancer, as reported in previous studies. Furthermore, inhibition of SGK1 has demonstrated potent anti‐tumor effects. Suppression of SGK1 has been associated with the induction of both autophagy and apoptosis. Differential gene expression analysis, visualized through a volcano plot, revealed a significant downregulation of SGK1 following treatment with 10% LDS (Figure 5C).

### 3.6 LDS Affects the Expression of Proteins Involved in Apoptosis and Autophagy Pathway in GBM Cells

**FIGURE 6:**
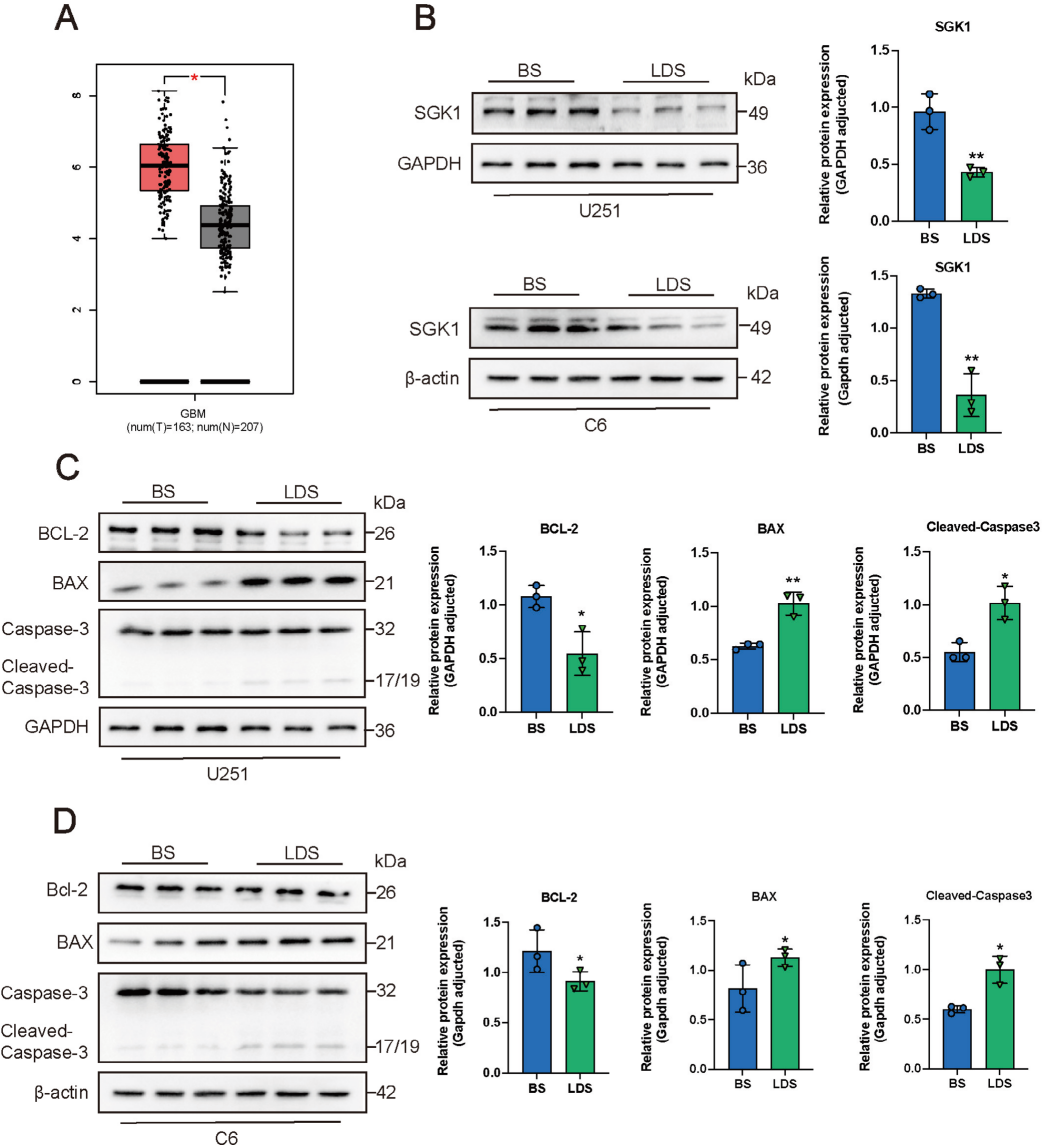
LDS affects the expression of proteins involved in apoptosis pathway in GBM cells. (A) Box plot of differential expression level in GBM tissue (red box) and normal tissue (gray box) on mRNA analyzed by GEPIA (http://gepia.cancer‐pku.cn). (B–D) SGK1 and proteins associated with apoptosis were examined by Western blotting in GBM cells treated with 10% BS (control group) and 10% LDS for 48 h. Expression of BAX was up‐regulated, expression of SGK1 (B), BCL‐2 was down‐regulated while ratios of cleaved Caspase‐3 were up‐regulated (C, D). Experiments were performed in triplicate. Data are expressed as mean ± SD (*n* = 3). *p < 0.05, **p < 0.01, ***p < 0.001 versus control. GBM, glioblastoma; LDS, leech drug‐containing serum.

**FIGURE 7:**
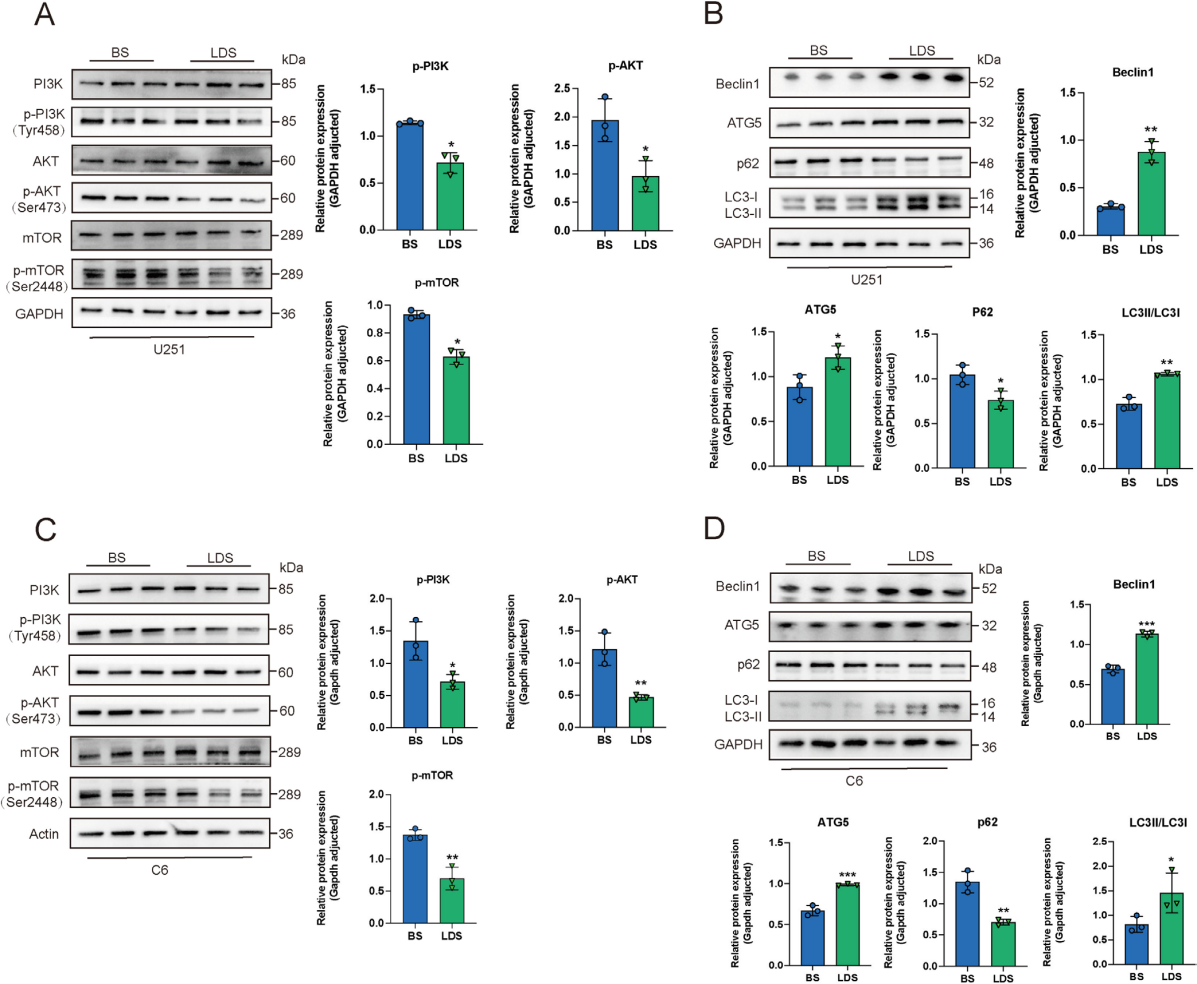
LDS affects the expression of proteins involved in the autophagy pathway in GBM cells. (A–D) Western blotting detection of proteins associated with autophagy and PI3K/AKT/mTOR in GBM cells treated with 10% BS (control group) and 10% LDS for 48 h. Expression of Beclin1, and ATG5 was up‐regulated, expression of p62 was down‐regulated while ratios of p‐PI3K, p‐AKT, p‐mTOR were down‐regulated and ratios of LC‐II/LC‐I were up‐regulated in U251 cells (A, B) and C6 cells (C, D). Experiments were performed in triplicate. Data are expressed as mean ± SD (*n* = 3). *p < 0.05, **p < 0.01, ***p < 0.001 versus control. GBM, glioblastoma; LDS, leech drug‐containing serum.

Based on data from GEPIA, our analysis revealed that SGK1 mRNA levels were elevated in tumor tissues compared to normal tissues (Figure 6A). Consequently, we proceeded to assess the expression of SGK1 proteins in U251 and C6 cell lines. After a 48‐h incubation period, LDS was observed to significantly downregulate SGK1 expression (Figure 6B). Subsequently, analysis of apoptosis‐related proteins indicated that LDS markedly decreased the expression of B‐cell lymphoma‐2 (BCL‐2) while increasing the levels of B‐cell lymphoma‐2‐Associated X (BAX) and Cleaved‐caspase 3 proteins (Figure 6C,D). SGK1 inhibitor GSK650394 inhibited the expression of SGK1 and enhanced the expression of BAX, indicating that LDS regulates apoptosis‐related proteins expression through SGK1(Figure S2). Prior research has demonstrated that LDS can induce autophagy in GBM cells. Subsequent transcriptome sequencing analysis indicated that treatment with LDS resulted in significant enrichment of the autophagy‐related PI3K/AKT and mTOR pathways, as revealed by KEGG analysis. Autophagy is a fundamental and conserved catabolic process that is crucial for a variety of cellular functions. The PI3K/AKT/mTOR pathway, a critical regulator of autophagy, is associated with the progression of various tumors [17, 18]. After a 48‐h treatment with 10% LDS, western blot analysis was employed to assess the protein levels of molecules associated with the PI3K/AKT and mTOR pathways in U251 and C6 cells. The results indicated that LDS inhibited the phosphorylation of PI3K, AKT, and mTOR signaling molecules in both U251 and C6 cells (Figure 7A,C). Furthermore, proteins involved in the downstream mTOR pathway, including Coiled‐coil myosin‐like BCL2‐interacting 19 protein (Beclin1), autophagy‐related 5 (ATG5), and sequestosome 1 (p62), were evaluated using western blot following the treatment of GBM cells with LDS. The results demonstrated that LDS significantly enhanced the expression of Beclin1 and ATG5 while inhibiting the expression of p62. Furthermore, it facilitated the conversion of the autophagy marker protein light chain 3 (LC3) from LC3‐I to LC3‐II (Figure 7B,D). These findings suggest that LDS induces autophagy in GBM cells by inhibiting the PI3K/AKT/mTOR pathway.

### 3.7 Leech Inhibits GBM Growth In Vivo

**FIGURE 8:**
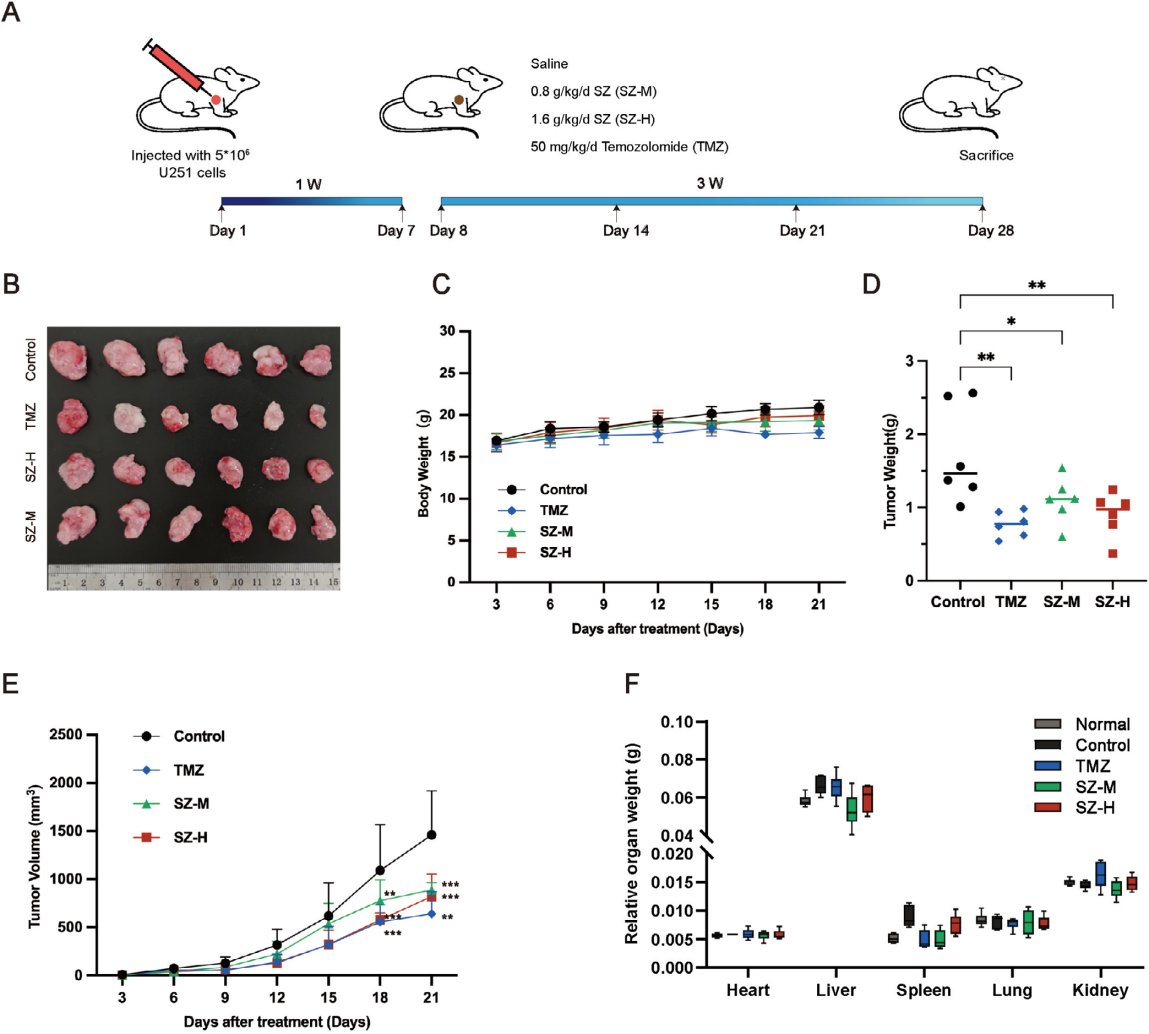
Leech suppresses GBM in vivo. (A) Schematic diagram of GBM xenograft mouse model treated with leech in vivo. (B) Image of tumors in different groups treated with LDS for 3 weeks. (C) Body weight during administration of LDS. (D) Tumor weight after 3 weeks of treatment with leech. (E) Tumor volume during administration of LDS. (F) Relative organ weights after 3 weeks of treatment with LDS. Data are expressed as mean ± SD (*n* = 6). *p < 0.05, **p < 0.01, ***p < 0.001 versus vehicle. GBM, glioblastoma; LDS, leech drug‐containing serum.

**FIGURE 9:**
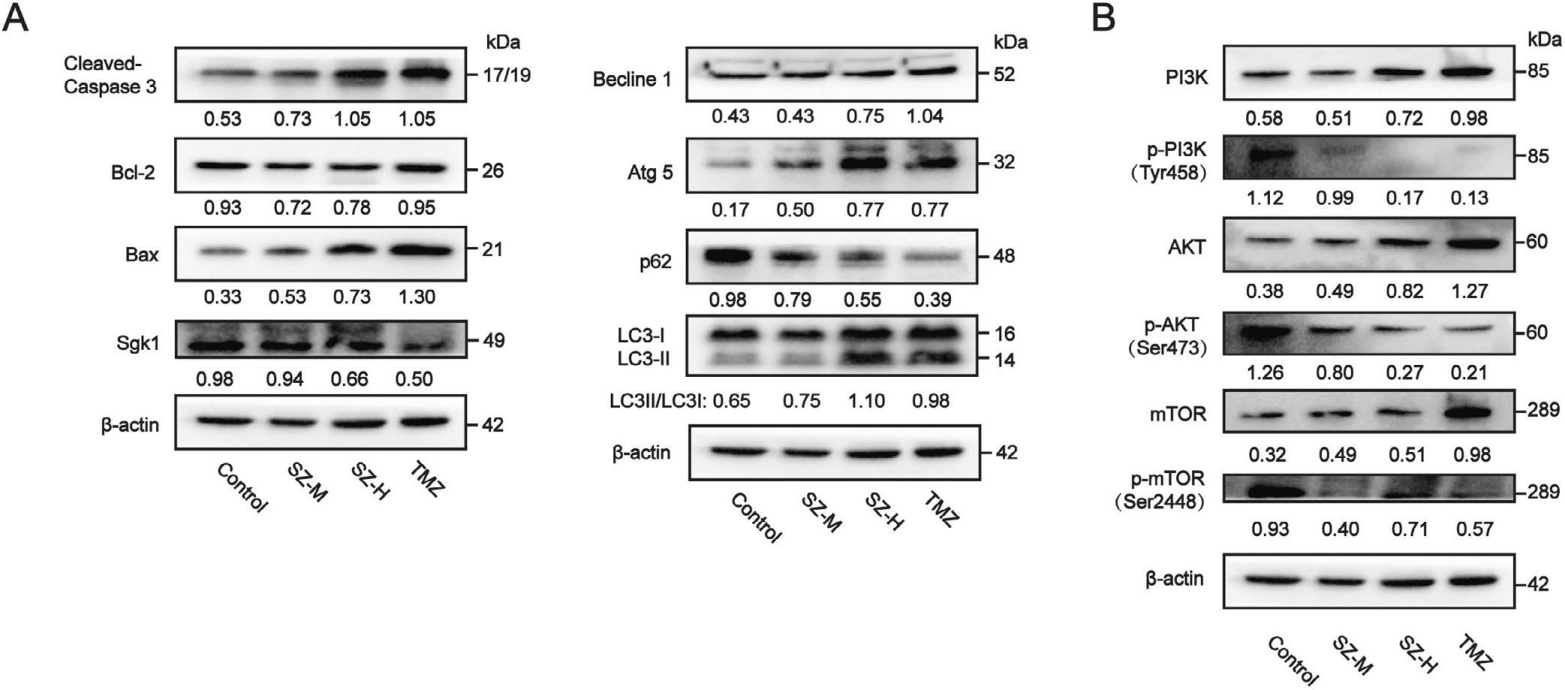
Leech influences protein expression of apoptosis and autophagy in GBM tissues. (A, B) Western blotting detection of SGK1 and proteins associated with apoptosis and autophagy (A) and PI3K/AKT/mTOR (B) in tumor tissues treated with leech drug. Expression of BAX was up‐regulated, expression of SGK1, and BCL‐2 was down‐regulated while ratios of cleaved Caspase‐3 were up‐regulated. Expression of Beclin1, and ATG5 were up‐regulated, expression of p62 was down‐regulated while ratios of p‐PI3K, p‐AKT, p‐mTOR were down‐regulated and ratios of LC‐II/LC‐I were up‐regulated. Experiments were performed in triplicate. Data are expressed as mean ± SD (*n* = 3). *p < 0.05, **p < 0.01, ***p < 0.001 versus control. GBM, glioblastoma.

To assess the effects of leech on GBM in vivo, a subcutaneous xenograft tumor model was developed in nude mice using U251 cells, as illustrated in Figure 8A. Nude mice were randomly assigned to four groups, comprising a control group, a positive control group (treated with TMZ), and two experimental groups receiving leech extract at doses of 80 mg/kg/day (SZ‐M) and 160 mg/kg/day (SZ‐H), respectively. The administration of leech ultramicro decoction pieces and TMZ was conducted over a period of 21 days. Throughout the study, changes in body weight and tumor size were systematically recorded. Upon conclusion of the treatment period, major organs and tumor tissues were harvested for analysis to evaluate the therapeutic efficacy of the leech ultramicro decoction pieces. Notably, the tumor volumes in the medium‐dose leech group (SZ‐M) and the high‐dose leech group (SZ‐H) were significantly reduced compared to the control group (Figure 8B). The tumor weight (Figure 8D) and growth rate (Figure 8E) in the leech drug‐treated group were significantly reduced, with the SZ‐H group demonstrating a tumor inhibitory effect comparable to that of the positive control group treated with TMZ. Notably, there was no significant change in body weight observed across all experimental groups (Figure 8C), and the treatment did not significantly affect the weight of major organs in the nude mice (Figure 8F). In conclusion, leech extract effectively inhibits the growth of GBM in murine models. We analyzed the proteins extracted from tumor tissues to further investigate the molecular mechanisms underlying the inhibition of GBM growth in vivo. The findings indicated that the leech‐derived compound significantly reduced the expression of SGK1 (Figure 9A) and modulated the expression of proteins associated with apoptosis and autophagy. Specifically, it downregulated BCL‐2 and P62, while upregulating Cleaved‐Caspase 3, BAX, Beclin1, and ATG5. Additionally, the compound facilitated the conversion of LC3 from LC3‐I to LC3‐II (Figure 9A). Concurrently, the leech compound markedly inhibited the phosphorylation of PI3K, AKT, and mTOR (Figure 9B). Collectively, these findings illustrate that leeches possess not only safety properties but also tumor‐suppressive capabilities in vivo. Furthermore, the results corroborate that leeches induce apoptosis and autophagy in GBM cells via the PI3K/AKT/mTOR signaling pathway. What's more, HE staining on major organs (Figure S3) showed that leech ultramicro decoction pieces had good biological safety.

## 4 Discussion

GBM represents the most aggressive and prevalent form of brain cancer [19, 20]. Currently, TMZ is the only approved chemotherapeutic agent for the treatment of GBM. However, it is reported that a significant proportion of GBM patients exhibit resistance to TMZ [21]. In this study, we discovered that the LDS dramatically suppressed colony formation, invasion, migration, and proliferation. Furthermore, LDS‐induced apoptosis and autophagy in GBM cells, thereby inhibiting GBM through the PI3K/AKT/mTOR signaling pathway.

Both traditional Chinese medicine (TCM) and Western medicine offer complementary perspectives and therapeutic approaches for understanding and managing health and disease [22]. As a holistic medical system rooted in Chinese philosophy, TCM is distinguished by its unique theoretical foundations and diagnostic methodologies. The integration of TCM with Western medicine represents a pivotal development in contemporary healthcare, and is poised to become an important paradigm in global medical practice [23]. Presently, enhancing patient survival is regarded as the foremost endpoint for evaluating the benefits of treatments for malignant tumors. While conventional therapies, including surgery, chemotherapy, radiotherapy, and targeted pharmaceuticals, have demonstrated a degree of efficacy, they are invariably associated with adverse reactions and complications, such as gastrointestinal symptoms and cardiotoxicity. Considering these challenges, TCM not only has the potential to ameliorate clinical symptoms but can also be integrated with Western medical treatments in a phased manner to mitigate adverse reactions [24, 25].

Nonetheless, the effectiveness of these medications requires further enhancement, and there is a clinical necessity for more Chinese medicines supported by robust evidence‐based medical research. The research and development of new drugs demand substantial investment and extended timelines. Therefore, for certain intractable diseases, it is imperative to establish a drug development strategy that can expedite the development process while minimizing costs and risks. “Old drugs for new therapeutic purposes” represents a cost‐effective and expedited strategy for addressing diseases beyond their original indications [26]. With advancements in high‐throughput screening technologies, computer‐aided drug design, and omics, the repurposing of established drugs has entered a new phase of development. The leech, a TCM with a long‐standing history, was first documented in Shennong's Classic of Materia Medica. It is traditionally used to dispel blood stasis, alleviate menstrual disorders, dissolve accumulations and masses, and address infertility [27]. Hirudin serves as a potent thrombin inhibitor and has been applied to a diverse range of human tumors, such as epithelioid carcinoma, adenocarcinoma, lung carcinoma, cholangiocarcinoma, and breast adenocarcinoma. It enhances the inactivation state of extracellular signal‐regulated kinase 1/2 (ERK1/2), downregulates the expression of the canonical mitogen‐activated protein kinase (MAPK)/ERK signaling pathway, and plays a pivotal role in the treatment of GBM. Recombinant hirudin, in conjunction with stealth liposomal vinblastine, has been shown to retard the proliferation of murine B16 melanoma cells and suppress pulmonary metastasis. This effect may be attributed to the inhibition of thrombin following recombinant hirudin administration, as well as the modulation of proteins associated with adhesion and angiogenesis, specifically focal adhesion kinase and vascular endothelial growth factor receptor. Furthermore, leech therapy may impede tumor angiogenesis by ameliorating the hypoxic microenvironment within tumors. This process is potentially facilitated by the reduction of hypoxia‐inducible factor‐1 alpha and vascular endothelial growth factor levels.

The serine/threonine kinase SGK1 was initially identified in mammary tumor cells and is a critical element of oncogenic signaling pathways associated with tumor proliferation, cellular transformation, and resistance to radiation and chemotherapy [28]. Although SGK1 inhibitors have shown potent anticancer effects by inducing G2/M cell cycle arrest and apoptosis, their therapeutic application is limited by several significant challenges [29]. Notably, many tumor cells exhibit elevated SGK1 expression levels. Furthermore, ectopic expression of SGK1 has been observed to inhibit apoptosis in the absence of growth factors, indicating its role as a survival kinase [30]. In this study, we provide evidence suggesting that SGK1 is a potential target gene for leech and other prospective therapeutic agents in GBM.

Our investigation revealed that the LDS treatment significantly induced apoptosis in human GBM cell lines, as evidenced by a decrease in Cleaved‐caspase 3/caspase 3 ratios and BCL‐2 expression, alongside an increase in BAX expression. These results corroborate the hypothesis that LDS initiates apoptosis [31]. Additionally, hirudin was observed to promote autophagy via the PI3K/AKT signaling pathway [32, 33]. Our findings indicate that LDS may induce autophagy in GBM through the PI3K/AKT/mTOR pathway. Nonetheless, we acknowledge the absence of a response experiment to validate our conclusions. So, we will proceed with further excavation in the subsequent study.

According to the findings of the RNA‐Seq research, LDS plays a critical role in modulating autophagy and apoptosis. Previous studies suggest that the repertoire of cellular regulatory proteins involved in autophagy and apoptosis is largely similar. For example, BCL‐2 and Bcl‐X(L) interact with and neutralize Beclin1, which contains the BCL‐2 homology 3 domain, thereby inhibiting autophagy and mitochondria‐mediated apoptosis by blocking pro‐apoptotic BCL‐2 homology 3 domain‐only proteins [34]. Both apoptosis and autophagy are significant processes in tumorigenesis [35]. Apoptosis is a crucial mechanism for maintaining physiological health by eliminating outdated, superfluous, and diseased cells. The process is characterized by three primary biochemical changes: the activation of caspases, the degradation of proteins and DNA, and alterations in cellular membranes that facilitate recognition by phagocytes [36]. Autophagy influences tumor cells through dual mechanisms. It acts as a preventive measure against cancer by removing defective organelles and misfolded proteins, thereby reducing oxidative stress within cells and ultimately preventing genetic damage. Moreover, SOCS5, a member of the suppressor of cytokine signaling (SOCS) protein family, plays a pivotal role in tumorigenesis. Research has shown that the overexpression of SOCS5 promotes tumor growth and invasion through autophagy, which is mediated by the PI3K/AKT/mTOR signaling pathway. Meanwhile, during the advanced stages of tumor development, autophagy may facilitate the survival of tumor cells under conditions of hypoxia or nutrient deprivation. It is important to note that autophagy can be inhibited by excessive or prolonged stress, potentially leading to the initiation of apoptosis under specific conditions. Consequently, the fate of individual cells may be anticipated or modulated through a comprehensive understanding of the interactions between autophagy and apoptosis, processes frequently observed in malignancies. The cellular mechanisms linking autophagy and apoptosis are presently under extensive investigation. These mechanisms, which encompass the interaction between BCL‐2 and Beclin‐1, as well as the regulation of signaling pathways by transcription factors and protein kinases, have the potential to influence both apoptosis and autophagy [37, 38].

Our experimental results demonstrate that leech‐derived compounds simultaneously induce apoptotic and autophagic pathways in tumor cells. These findings suggest a potential crosstalk between these two cellular processes. Further investigation is required to elucidate the temporal and mechanistic relationship between apoptosis and autophagy activation. Notably, the initiation of programmed cell death appears to be dependent on both the magnitude and duration of cellular stress exceeding homeostatic thresholds. Upon initiation of the apoptotic pathway, caspases are activated, which cleave various proteins to facilitate apoptosis. Concurrently, caspases degrade several key autophagy‐related proteins, such as ATG3 and Beclin 1, thereby inhibiting autophagy, disrupting the cell's self‐protective mechanisms, and expediting cell death. Furthermore, certain autophagy protein fragments generated through caspase‐mediated cleavage exhibit pro‐apoptotic properties. Specifically, Beclin 1, a pivotal regulatory protein in autophagy, undergoes cleavage by caspase 3, caspase 6, or caspase 9. This cleavage facilitates the generation of a carboxyl‐terminal fragment downstream of the BH3 domain of the pro‐apoptotic protein BCL‐2, thereby promoting the release of cytochrome C and ultimately culminating in apoptosis [39]. In certain specific contexts, the induction of autophagy can activate the apoptotic pathway. According to recent studies, the inhibition of autophagy during its initial stages leads to a reduction in the activities of caspases 8 and 3. Conversely, the suppression of autophagy in its later stages results in an increase in caspase‐dependent cell death. Consequently, researchers hypothesize that the formation of autophagosomes may facilitate the activation of caspases [40]. Further investigation is necessary to elucidate the relationship between apoptosis and autophagy in the context of leech treatment.

In vivo studies demonstrate that leech therapy effectively inhibits the progression of GBM while maintaining a favorable safety profile. Administration of leech‐derived compounds at dosages of 160 and 80 mg/kg significantly suppressed GBM growth. Mice treated with leech extracts exhibited no noticeable signs of dermatitis or gastrointestinal disturbances. In summary, our study demonstrates that leech‐derived compounds exhibit significant anti‐glioblastoma activity through dual induction of apoptotic and autophagic pathways. These findings not only reveal a novel therapeutic mechanism involving coordinated SGK1 inhibition and autophagy activation in GBM treatment, but also provide compelling evidence for developing combination therapies targeting these synergistic pathways to overcome therapeutic resistance.

The subcutaneous xenograft tumor model (used in the current study) did not mimic the effect of the blood–brain barrier (BBB) on drug penetration and cannot assess the invasive properties of the tumor to the surrounding brain tissue. The PDOX model (Patient‐Derived Orthotopic Xenograft Model) could be used to confirm our results in future research. Although our results showed that LDS inhibited the growth of GBM cells by promoting autophagy and inducing apoptosis in vitro and in vivo, the components of LDS are complex and the truly effective components need to be further explored. Leeches show good anti‐GBM effects, but their therapeutic efficacy is not as good as the clinical first‐line drug temozolomide. Whether the combination of leeches and temozolomide can enhance the efficacy also deserves to be explored.

## 5 Conclusion

The leech‐derived compounds exhibited a good anti‐tumor activity against glioblastoma. The LDS inhibited the proliferation, migration and invasion of glioblastoma cells. Mechanistically, the leech‐derived compounds downregulated the expression of SGK1 and induced apoptosis and autophagy which may be caused by the modulation of SGK1/Caspase‐3 and PI3K/AKT/mTOR pathways. In conclusion, the leech holds potential as a promising therapeutic agent for the treatment of GBM.

## Author Contributions

Shaohua Wu: writing – original draft, validation, methodology, data curation, conceptualization. Yaya Zhou: writing – review and editing, validation, methodology, formal analysis. Zhuan Pei: validation, methodology. Yang Wang: conceptualization, resources, supervision, project administration. Zuping Zhang: writing – review and editing, conceptualization, resources, funding acquisition. All the authors determined the concept and scope of the article.

## Ethics Statement

All animal procedures were approved by the Committee on the Care and Use of Animals at Central South University (SYXK‐2020‐0019).

## Conflicts of Interest

The authors declare no conflicts of interest.

## Supporting information


**Figure S1:** Qualitative analysis of blank serum (BS) and leech drug‐containing serum (LDS). The serum samples were prepared in a solvent mixture of 0.1% methanol/water and 0.1% formic acid/acetonitrile. Separation was performed on an LCMS‐8050 system with a flow rate of 0.4 mL/min and an injection volume of 10 μL. compounds were ionized in negative mode using a triple quadrupole mass spectrometer. (A) Analysis of BS compounds. (B) Analysis of LDS compounds.
**Figure S2:** LDS regulates BAX expression through SGK1. SGK1 and BAX proteins expression were detected by Western blotting in U251 cells treated with 10% BS, 10% LDS, 10% BS + GSK650394 (SGK1 inhibitor), 10% LDS + GSK650394 (SGK1 inhibitor).
**Figure S3:** HE staining on heart, liver, spleen, lung and kidney of mice in control, SZ‐M, SZ‐H and TMZ groups.

**Data S1:** cns70683‐sup‐0002‐DataS1.pdf.

## Data Availability

All data that support these findings of the study are available on request from the corresponding author upon reasonable request.
