# Supplementary figures and images for: Traditional Chinese Medicinal Leech Induces Apoptosis and Autophagy in Glioblastoma by SGK1/Caspase‐3 and PI3K/AKT/mTOR Pathway
Source: CNS Neurosci Ther. 2025 Dec 1;31(12):e70683. doi: 10.1002/cns.70683 (PMC12668900; doi:10.1002/cns.70683)

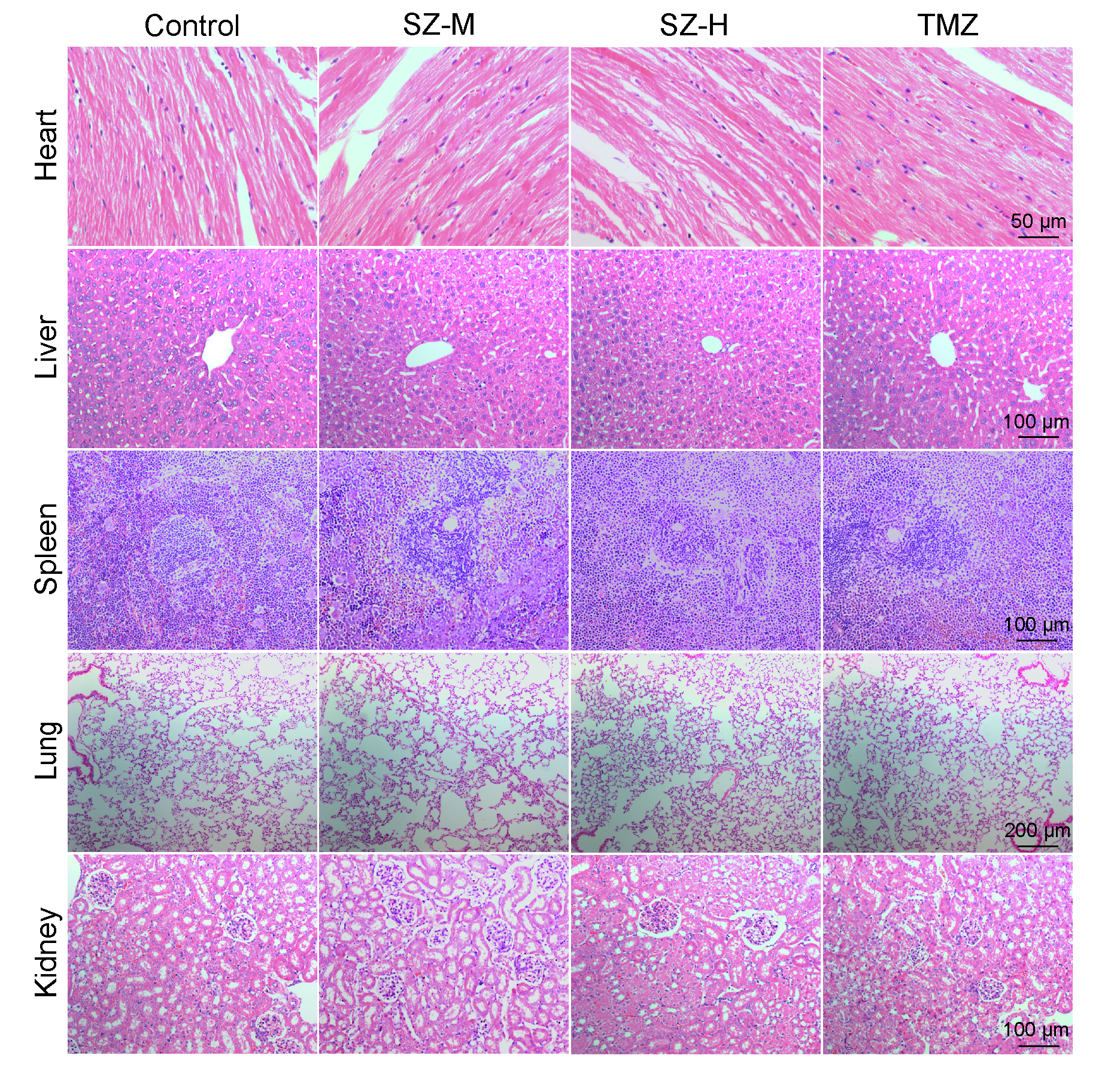

Supplement: Supplementary file 1 — Figure S1: Qualitative analysis of blank serum (BS) and leech drug‐containing serum (LDS). The serum samples were prepared in a solvent mixture of 0.1% methanol/water and 0.1% formic acid/acetonitrile. Separation was performed on an LCMS‐8050 system with a flow rate of 0.4 mL/min and an injection volume of 10 μL. compounds were ionized in negative mode using a triple quadrupole mass spectrometer. (A) Analysis of BS compounds. (B) Analysis of LDS compounds. Figure S2: LDS regulates BAX expression through SGK1. SGK1 and BAX proteins expression were detected by Western blotting in U251 cells treated with 10% BS, 10% LDS, 10% BS + GSK650394 (SGK1 inhibitor), 10% LDS + GSK650394 (SGK1 inhibitor). Figure S3: HE staining on heart, liver, spleen, lung and kidney of mice in control, SZ‐M, SZ‐H and TMZ groups. [file CNS-31-e70683-s001.zip › Figure S3.tif]

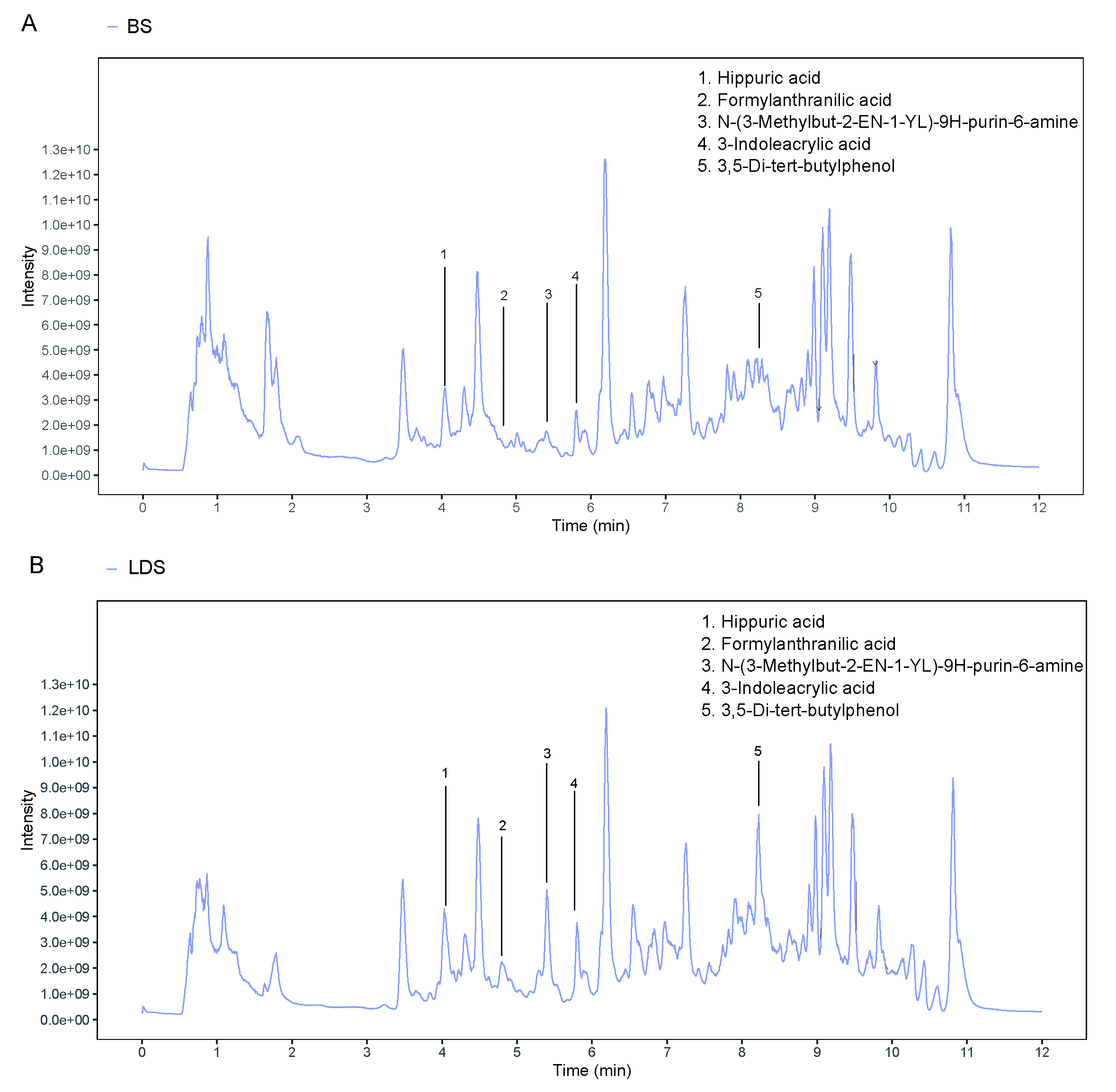

Supplement: Supplementary file 1 — Figure S1: Qualitative analysis of blank serum (BS) and leech drug‐containing serum (LDS). The serum samples were prepared in a solvent mixture of 0.1% methanol/water and 0.1% formic acid/acetonitrile. Separation was performed on an LCMS‐8050 system with a flow rate of 0.4 mL/min and an injection volume of 10 μL. compounds were ionized in negative mode using a triple quadrupole mass spectrometer. (A) Analysis of BS compounds. (B) Analysis of LDS compounds. Figure S2: LDS regulates BAX expression through SGK1. SGK1 and BAX proteins expression were detected by Western blotting in U251 cells treated with 10% BS, 10% LDS, 10% BS + GSK650394 (SGK1 inhibitor), 10% LDS + GSK650394 (SGK1 inhibitor). Figure S3: HE staining on heart, liver, spleen, lung and kidney of mice in control, SZ‐M, SZ‐H and TMZ groups. [file CNS-31-e70683-s001.zip › Figure S1.tif]

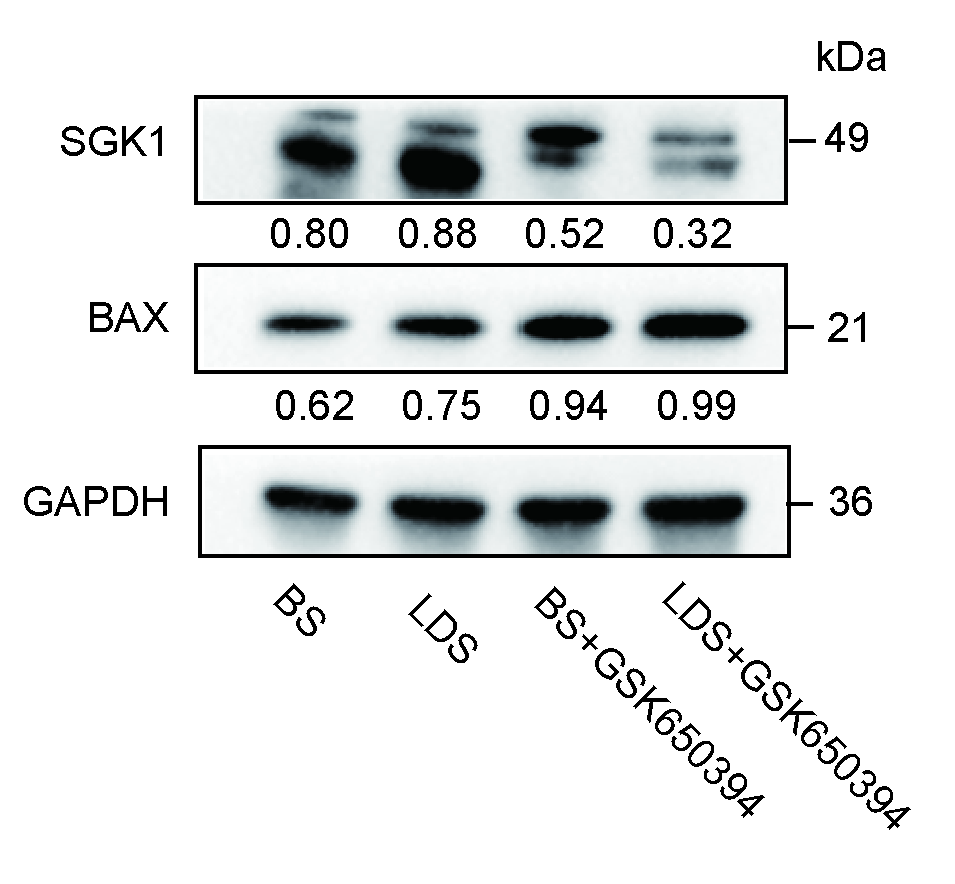

Supplement: Supplementary file 1 — Figure S1: Qualitative analysis of blank serum (BS) and leech drug‐containing serum (LDS). The serum samples were prepared in a solvent mixture of 0.1% methanol/water and 0.1% formic acid/acetonitrile. Separation was performed on an LCMS‐8050 system with a flow rate of 0.4 mL/min and an injection volume of 10 μL. compounds were ionized in negative mode using a triple quadrupole mass spectrometer. (A) Analysis of BS compounds. (B) Analysis of LDS compounds. Figure S2: LDS regulates BAX expression through SGK1. SGK1 and BAX proteins expression were detected by Western blotting in U251 cells treated with 10% BS, 10% LDS, 10% BS + GSK650394 (SGK1 inhibitor), 10% LDS + GSK650394 (SGK1 inhibitor). Figure S3: HE staining on heart, liver, spleen, lung and kidney of mice in control, SZ‐M, SZ‐H and TMZ groups. [file CNS-31-e70683-s001.zip › Figure S2.tif]

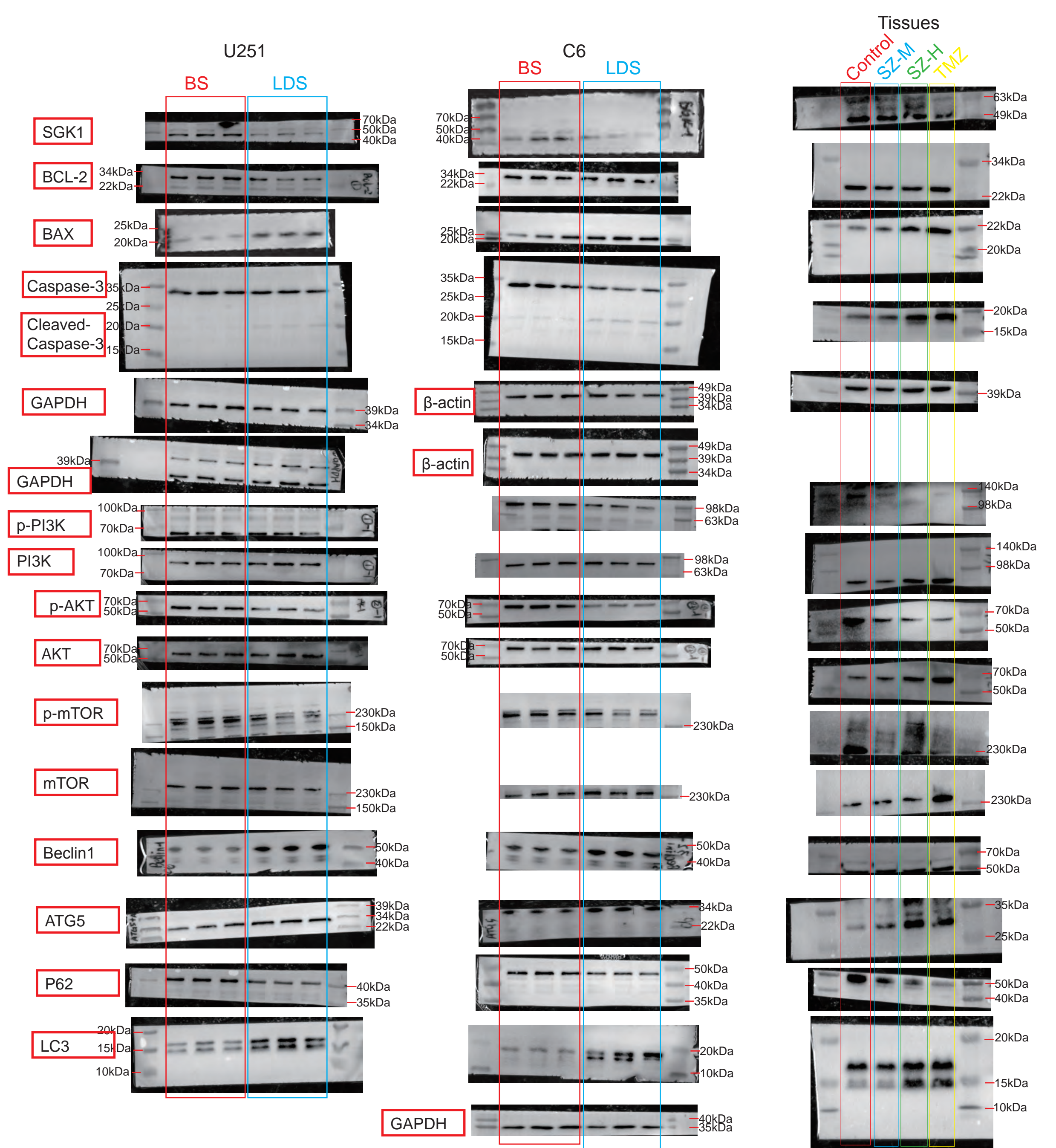

Supplement: Supplementary file 2 — Data S1: cns70683‐sup‐0002‐DataS1.pdf. [file CNS-31-e70683-s002.pdf]
